# A study on real-time low-quality content detection on Twitter from the users’ perspective

**DOI:** 10.1371/journal.pone.0182487

**Published:** 2017-08-09

**Authors:** Weiling Chen, Chai Kiat Yeo, Chiew Tong Lau, Bu Sung Lee

**Affiliations:** School of Computer Science and Engineering, Nanyang Technological University, Singapore, Singapore; University of Cape Town, SOUTH AFRICA

## Abstract

Detection techniques of malicious content such as spam and phishing on Online Social Networks (OSN) are common with little attention paid to other types of low-quality content which actually impacts users’ content browsing experience most. The aim of our work is to detect low-quality content from the users’ perspective in real time. To define low-quality content comprehensibly, Expectation Maximization (EM) algorithm is first used to coarsely classify low-quality tweets into four categories. Based on this preliminary study, a survey is carefully designed to gather users’ opinions on different categories of low-quality content. Both direct and indirect features including newly proposed features are identified to characterize all types of low-quality content. We then further combine word level analysis with the identified features and build a keyword blacklist dictionary to improve the detection performance. We manually label an extensive Twitter dataset of 100,000 tweets and perform low-quality content detection in real time based on the characterized significant features and word level analysis. The results of our research show that our method has a high accuracy of 0.9711 and a good F1 of 0.8379 based on a random forest classifier with real time performance in the detection of low-quality content in tweets. Our work therefore achieves a positive impact in improving user experience in browsing social media content.

## Introduction

Online Social Networks (OSN) in a web 2.0 era have developed from monotonous social interactions and communication into an integration of social media functions for all kinds of services [1]. In the last decade, more and more social network sites have sprung up and attracted millions of users. Among them, Facebook, QQ, Twitter are the most popular ones, with 1,590 million, 853 million and 320 million active users respectively as of April 2016 [2].

With the fast growth of OSN, they have become the new target of many cyber criminals like spammers and phishers as well as many advertisers which have resulted in worrying issues. Spam is usually designed to make the potential victims spend money on fake or counterfeit products and services or are just outright frauds [3]. Botnets and virus-infected computers are commonly used to send the majority of spam messages, including job-hunting advertisements, promotions of free vouchers, testimonials for some pharmaceutical products, etc [4]. Phishing can be recognized as a special type of spam that is intended to trick the recipients into revealing their personal information especially sensitive data like login and password details. After obtaining the personal or account information, the phishers can breach the victims’ accounts and commit identity theft or fraud.

According to Networked Insights’ research, as of fall 2014, 9.3% of content on Twitter are spam [5]. Apart from these spam and phishing content, the OSN also suffer from large amount of low quality content including advertisements, automatically generated content by third party applications, etc. Users are hampered from browsing meaningful and interesting content by the overwhelming amount of low-quality content, resulting in significant decrease in the overall user experience of using the OSN. In some extreme cases, they can even affect the physical condition of some vulnerable users with a syndrome called “Twitter psychosis” [6].

Researchers have paid much attention to the detection of malicious content such as phishing or spam while in contrast little attention has been given to the large quantity of repeated low-quality content which bothers users most. Very few of the previous work are carried out from the users’ perspective. Thus it is important to develop a unified technique to filter all these low-quality content so as to improve the overall user experience instead of focusing on spam or phishing alone. Herein lies the motivation for the research carried out in this paper. The reason that we are using the term “low-quality content” instead of the more familiar term “spam” is because the definition for spam is diverse and are often used to indicate malicious content. However, in our case, these malicious content only accounts for a small proportion of all low-quality content. In other words, there are other types of low-quality content besides spam. Hence, to avoid the potential misunderstanding, we will use the term low-quality content instead of spam.

Considering that there is not a general consensus about the definition of low-quality content on OSN, this adds to the difficulties of detection as well as the evaluation of different detection methods. A further question is whether the features selected for detecting spam or phishing can still be efficient when detecting other types of low-quality content. In addition, even if these features can achieve a high detection rate, can they be extracted in real time? The consideration is once a tweet is posted, it will be delivered to all the followers immediately. Thus, the real-time requirement is quite necessary for protecting and improving user experience when they are using the OSN. As a matter of fact, many of the current detection work is done offline. Graph features adopted in [7] such as betweenness centrality and the redirection information adopted in [8] are too time consuming, making it difficult to apply them in an online context. [9] also consumes much time when calculating the carefulness of users’ behaviors.

Different from existing research work whose attention is focused on the detection of malicious content such as spam and phishing messages, the research objective of our work is the detection of low-quality content on OSN which has a wider range than just malicious content. Another highlight we would like to emphasize is that the proposed features performed to characterize low-quality content are time efficient to compute which facilitates the real-time detection once the offline training is completed.

We carry out a survey to investigate user opinions about low-quality content and based on the survey results, we provide a clearer definition from the users’ perspective. Then we propose some features for low-quality content detection and verify whether the features frequently used for the detection of spam/phishing still apply for all types of low-quality content. Both the detection rate and time performance are adopted as the evaluation metrics so as to fulfill the requirement of an online environment.

In summary, our main contributions are as follows:

- We perform EM algorithm on harvested low-quality content tweets to divide them into 4 categories. Based on which, we create a survey with 211 participants to study their opinions about low-quality content. We then provide a clearer definition of low-quality content on Twitter according to the survey results. We are the first to carry out such preliminary studies from the users’ perspective.
- We crawl and manually label 100,000 tweets so as to verify the accuracy of our definitions and classification results. Example tweets and labeling guides are provided so as to make the experiments replicable.
- We believe the detection techniques for malicious content on OSN are quite mature but little attention is paid to other types of low-quality content such as low quality advertisements and automatically generated content which actually bothers users most. Thus we unify the detection of different types of low-quality content and provide an in-depth study of the features commonly used for detection of malicious content to understand their applicability for other low-quality content.
- We provide a word level analysis on original tweet texts and build a keyword blacklist dictionary to facilitate low-quality content detection. We are the first to build the dictionary by ourselves to help detect low-quality content.
- We apply traditional classifiers (SVM and random forest) based on our proposed dominant features as well as word features for real-time low-quality content detection and it achieves a high accuracy and F1 as well as a good time performance.

The remainder of the paper is organized as follows. We first discuss the related work pertaining to spam and phishing detection followed by the introduction of the overview of the proposed low-quality content detection system. Then we present the results of the survey we conducted and define the low-quality content in a clearer way based on the survey results. Thereafter, we provide a detailed study of features used for real-time low-quality content detection from the perspective of both time and accuracy. This is followed by a description of how we process and extract the various features from the original tweets. Then we illustrate the detection results using selected features and discuss the comparisons with other research work. The last section concludes the paper and presents the future work.

## Related work

In the last decade, the growth of online social networks has provided a new hotbed for spammers and phishers. Significant efforts have been paid to detect and analyze the malicious content on social websites like Facebook, Twitter, etc.

### Definition of low-quality content

Spam on OSN (sometimes called as social spam) is usually regarded as a message which is unsought for by legitimate users [10]. However “unsought” is quite a vague description. Different research work have different definitions for spam and phishing. Yang et al. regard tweets which post malicious content as spam and does not consider advertisements [7]. Thomas et al. [11] and Sridharan et al. [12] label a tweet as spam if the account is suspended by Twitter in a later validation request. However, the definition in [7] is closer to that of phishing instead of spam while [11] and [12] also have drawbacks as they use Twitter suspension policy as a reference. Twitter itself initially only focused on spam or phishing according to Twitter Rules [13] while showing generosity to mainline bot-level access and some advertisements as long as they do not break Twitter rules [14]. Currently, Twitter has introduced a quality filter recently which aims to filter out low-quality content [15]. This testifies to the usefulness of our work. It is to be noted that Twitter’s quality filter is applied on the notification timeline (i.e. tweets mentioning the user) while our work is applied on the users’ home timeline (i.e. all the users’ friends’ tweets). In other words, only tweets mentioning the user will be processed by Twitter’s quality filter while our method does not have such limitations.

From Twitter policy, we can see that accounts which persistently post low-quality content are less likely to be suspended. Moreover, account suspension may not only be due to the delivery of spam, thus making the judging yardstick even less convincing. One thing in common among these definitions is that they try to characterize the features or behaviors of these unsolicited content themselves instead of defining them from the users’ perspective. In addition, not much work is focused on low-quality content detection. They either focus on simplex spam or phishing detection instead of proposing a unified detection technique which also aims at other low-quality content. Lee et al. first propose the term “content polluters” and divides them into several categories [16]. However, in their work, the term “content polluter” is used to refer to spam accounts while we use “low-quality content” to refer to tweets which contain only valueless and trivial content. The difference between their work and ours actually reflects two mainstream research ideas which will be introduced in the next subsection.

### Prevalent detection techniques

Spam or phishing detection is often regarded as a classification problem. Some research work put more efforts into the detection of spam accounts [17] [18] [19] while others shift the focus to the detection of spam tweets [20] [21]. Regarding the two different detection methods, a lot of attention is focused on selecting the most significant features. These features can also be divided into two categories, namely, user based and tweet based.

Lee et al. systematically divide user based features into four groups for the first time [7]. The authors adopt four feature sets, including User Demographics (UD), User Friendship Networks (UFN), User Content (UC) and User History (UH). Then they use different classifiers to perform spammer account detection based on their proposed features.

Yang et al. also propose some social based features like number and ratio of bi-directional links, betweenness centrality (BC) as well as clustering coefficient (CC) for spam detection [7]. However their features are time-consuming to calculate making it less feasible for real-time detection. Moreover, Ferrara et al. describes user behaviors that best discriminate social bots from humans [22] while Grier et al. concludes that the behaviors of bots (spammers) are often less complex than that of humans [23]. Almaatouq et al. further analyze the social interactions between users including the follow and mention relationships [24]. In a more recent work [25], they focus on the detection of bots on Twitter using similar features mentioned before like network features, temporal behavior features, etc.

Tweet based features can usually be divided into three groups. They include tweet content, tweet sentiment and tweet semantics. Tweet content features are usually calculated by counting the number of specific words, symbols or punctuation in tweets [14]. Tweet sentiment features are calculated by using some sentiment lexicons plus sentiment analysis [26]. Tweet semantics features exploit the natural language processing techniques to facilitate the low-quality content detection on OSN. Martinez-Romo et al. first use language as the primary tool for spam tweet detection [27]. The authors use the concept of Kullback-Leibler Divergence and extract three language model based features. Santos et al. apply compression-based text classification methods to avoid good words attack and improves detection performance for spam tweets [28]. Yang et al. extracts text information from both the web pages and the tags [29]. Then the authors measure the relatedness between the two so as to detect tag spam.

A variation of tweet based spam detection is the detection of spam campaigns [30] [31]. Gao et al. cluster tweets with common URLs together [32]. If these URLs are recorded in some blacklists such as GoogleSafe Browsing, PhishTank, etc, the cluster will be regarded as a spam campaign. This clustering method is not very applicable for low-quality content detection as some low-quality content like messy codes do not actually have links in them. Furthermore, to cluster tweets, we have to wait for similar tweets to accumulate up to a certain number making it less feasible for real time detection.

What is worth mentioning here is the term “low-quality content” used in this paper should be distinguished from “content polluter” used in Lee’s paper [16]. In their work, the term “content polluter” is more similar to a spammer’s account whereas we refer to low-quality content as a piece of **tweet message** of little value or importance to the users and may erode the users’ experience. The intuition behind is that even a normal user may post low-quality content with or without intention and a spammer may also post normal messages to avoid the potential suspension by Twitter. Our experimental results show a better performance can be achieved with fine-grained detection based on tweets instead of users.

### Real-time detection

Some research work have focused on real-time requirement for the detection of spam and phishing. Aggarwal et al. develops a plugin for browsers so as to implement the automatic real-time phishing detection on Twitter [33]. Song et al. further exploit the sender-receiver relationship [34]. When a user receives a message from a stranger, the system can identify the sender at once therefore ensuring that the clients can identify the spammers in real time. Tan et al. propose a runtime spam detection scheme known as BARS (Blacklist-assisted Run-time Spam Detection) which exploits the different behavioral patterns between normal users and spammers as well as a spam URL blacklist [35]. A more recent piece of work [14] exploits the inherent features of Twitter. All the features they use can be directly extracted or calculated which meets the real time requirement. However, again the aforementioned studies either focus on spam detection or phishing detection but do not provide a unifying solution for all low-quality content detection especially for the large amount of low quality advertisements, meaningless messy codes, etc.

Different from existing research work, we carry out a survey and study low-quality content from the users’ perspective for the first time. A clear definition of low-quality content is thus provided to facilitate further detection work. Then we test the features used in traditional spam or phishing detection to see whether they are still applicable to our new context and we characterize the most significant ones. Thus our work is valuable in filling the gaps of the current detection methods for low-quality content on online social networks and finally improving the user experience.

## Overview of the low-quality content detection system

**Fig 1:**
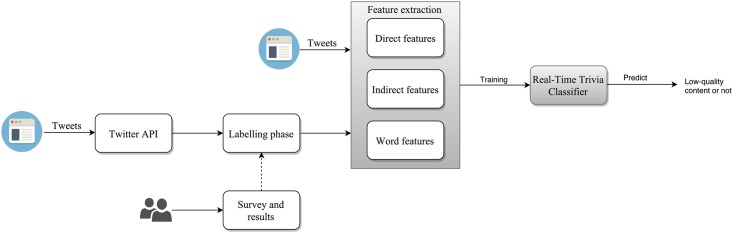
Overview of the low-quality content detection system.

Fig 1 shows the overview of our proposed low-quality content detection system. Our work comprises two portions, the actual real-time detection of low-quality content tweets (refer to the shaded blocks in Fig 1) and the out-of-band training process (refer to the unshaded boxes of Fig 1).

The training process is conducted out-of-band to train the classifier used for real time low-quality content detection. To be more specific, a user survey is conducted to provide insights on the definition of low-quality content from the users’ perspective. These are then used as label guides for manually labeling 100,000 tweets crawled via Twitter API. Significant features (both direct and indirect) of low-quality content are identified from the 100,000 labeled tweets and these features are combined with word level analysis to train the classifier.

After training the classifier, the classifier is ready to predict the labels of tweets submitted to our system. These tweets experience the same feature extraction phase as the training phase and are then forwarded to the trained classifier for low-quality content detection. It will then predict whether the tweet is low-quality content or not. What is worth mentioning here is that both the feature extraction and low-quality content detection can be done in real time.

## A study on low-quality content from users’ perspective

### Cluster analysis of low-quality content

We believe it is necessary to understand users’ attitudes and definitions of low-quality content before proceeding with the subsequent research. In order to design a survey which can fully convey users’ opinions about low-quality content, we manually investigated and verified low-quality content via cluster analysis.

The Streaming API provided by Twitter give developers low latency access to Twitter’s real-time global stream of tweet data. We use Streaming API to crawl 10,000 tweets as a preliminary dataset. Then three annotators are asked to label the tweets as either low-quality content or normal tweets in the dataset. During this phase, we only provide some general descriptions of low-quality content instead of clear labeling guidelines. If any of the three annotators marks the tweet as low-quality content, it will be regarded as potential low-quality content.

We admit there may be bias due to the limited size of the preliminary dataset and the three annotators’ opinions may not represent each and every user. However, labeling during this phase does not need to be that accurate and we only want to get a general idea of the low-quality content from users’ perspective so as to design the questions in the survey such that they are more typical and representative.

To perform the cluster analysis, we represent the tweets with a set of features (described in details in the later section) and then apply the Expectation Maximization (EM) algorithm [36] to group together tweets which have similar characteristics or behaviors. What inspires us to use EM algorithm to roughly classify the low-quality content is [16] which uses EM to group content polluters into several categories. However, the difference between our work and theirs is that we use EM to classify the tweets (i.e. low-quality content) instead of accounts (i.e. content polluters).

After removing groups with too few tweets and emerging groups with similar tweets, all these low-quality content can be divided into four categories:

- Low quality advertisements: These advertisements include not only deceptive or false advertisements but also those valueless advertisements posted by those obscure users. Two relative examples are “*Hot, my little pony friendship city light curtain. (hm118)—Full read by eBay (URL omitted)*” and “*take free bit-coin every three minute (URL omitted)*”. Some pornographic and violent content also appear in the form of advertisements which mars users’ experience when browsing normal tweets.
- Automatically generated content: These content are usually posted by some applications or online services instead of users themselves, mostly for promotion purposes. Once the user has given authorization to these applications and services, some user behaviors may trigger automatically generated content like “*I’ve collected 7,715 gold coins! (URL omitted) #android, #androidgames, #gameinsight*” or “*Today stats: 4 followers, No unfollowers via (URL omitted)*”. Content produced by the same application tends to be similar or has limited variations. A large amount of repetitive content significantly erodes the user experience.
- Meaningless content: Some of the meaningless content is also posted by bots and has different forms. Some of them are readable like quotes of famous people or the created time of the tweet. Some of them are unreadable like mere messy codes (e.g. g7302t$u!7#52jgi4o).
- Click baits: The characteristics of low-quality content falling into this category is not very obvious and they cover a wide range of topics. Many of them look like normal messages but in many cases, the link appearing in the tweet is not related to the tweet text. Furthermore, some of the links lead to malicious sites.

The screen captures of the different categories of content polluters can be seen in S1 Fig.

### Design of the survey

We designed a survey according to the cluster analysis and put it online where participants had to answer two questions related to personal information, namely, age and gender and eight questions related to online social networks and low-quality content. A full version of survey is shown in S1 Text. We are interested mainly in:

- The effects of low-quality content on user experience when using OSN.
- What kinds of content are regarded as low-quality content by users.
- To what degree can users tolerate low-quality content before considering unfollowing.

The survey is posted online and is entirely anonymous. At the beginning of the survey, the participants are told that the survey is anonymous and their responses will be used for research purpose. In other words, consent is implicit as in by taking part in this survey, it means the participant has given their consent. It was opened to anyone online and participants voluntarily participate in the survey. Hence no participant is harmed physically and mentally.

After posting the survey online, we have received 211 responses. All of them are valid as all questions in the survey are compulsory and the participant has to complete all questions in the survey then he or she can submit it.

As the survey link is posted on several famous online social websites (e.g. Twitter, Sina Weibo, etc), it ensures the survey results are indeed from OSN users. 88.7% of the respondents use OSN every day and 9.48% of them use OSN at least once a week. These participants are from different age groups with 74.88% in the 18 to 25 age group and 44.55% of them are females.

### Results of the survey

For this analysis, we focus on several aspects of low-quality content from the users’ perspective. First, we want to demonstrate the significance of our work. Technically, the filtering mechanism can be applied on both the Twitter server side and the client side. The fact is that Twitter only bans the accounts pertaining to abusive behaviors [13]. For obvious commercial reasons, Twitter will not ban advertisements unilaterally from its side, especially those repetitive low quality advertisements. For other low-quality content, as long as it does not violate Twitter rules, there is no reason for Twitter to filter them as well. However, too much such valueless content hampers users from browsing other meaningful content and 97.16% of participants believe such content affects their user experience more or less as shown in Table 1. Considering this, a filter applied on the client side will be very meaningful to the users.

**Table 1:** How much do content polluters affect your user experience when using social network sites?

Options	Number	Ratio
Very much.	48	22.75%
A bit but still bearable.	141	66.82%
A little.	16	7.58%
They don’t affect my user experience.	6	2.84%

To better understand the impact on users, we also ask participants about their responses to such low-quality content. One conjecture is that if one account posts too much low-quality content, its followers may tend to unfollow him or her. To our surprise, contradicting this conjecture, 72.51% of the respondents seldom or never clean up their followees (i.e. friends) although nearly 90% of them admit that low-quality content does affect their user experience more or less as shown in Table 1.

As shown in Table 2, 19.91% of the participants admit that, out of courtesy, they will follow reciprocally if someone follows them. The result corresponds to that in [37] indicating that users tend to reciprocate out of social etiquette.

**Table 2:** If someone follows you, will you follow back?

Options	Number	Ratio
follow back out of courtsey	42	19.91%
follow those I know or share common interests	149	70.62%
don’t follow back	15	7.11%
others	5	2.37%

**Fig 2:**
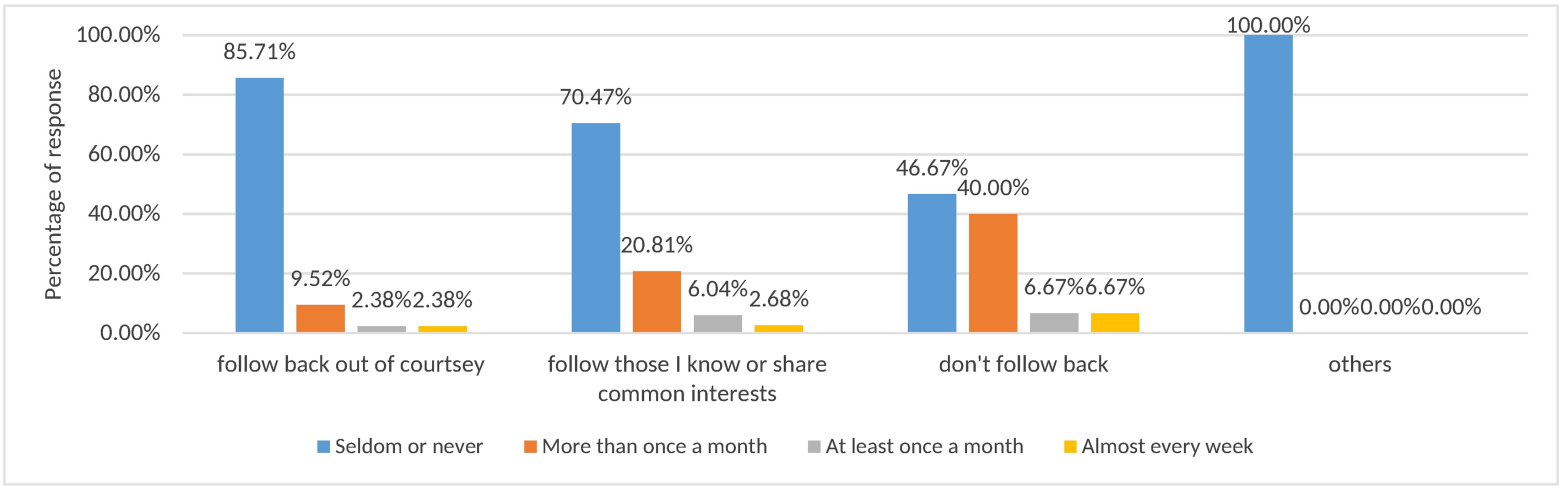
Users’ habits about following and cleaning up friends.

We then carry out a cross analysis for users’ habits about following (Table 2) and cleaning up friends (Table 3). The results are shown in Fig 2. Among the 19.91% of people who easily follow back, 85.71% of them actually do not clean up their friends regularly.

**Table 3:** How often will you clean up your followees/friends?

Options	Number	Ratio
Seldom or never.	153	72.51%
More than once a month.	41	19.43%
At least once a month.	11	5.21%
Almost every week.	6	2.84%

This finding is consistent with [38] that users tend to interact with only a small subset of their friends and pay little attention to as much as half of their friends. This further indicates that once a user who posts a lot of low-quality content tricks a normal user into following him in some way, it is highly possible that this user may continuously deliver low-quality content and affect others’ user experience because most users feel too bothersome to clean up the followees. Therefore, some users may have a close friendship with spammers. [39] makes similar conclusions in their research. Thus, it is necessary to set up an automatic filtering mechanism for low-quality content to improve the user experience of using OSN. It is to be noted that from Table 2, only 5 out of 211 respondents chose “Others” as their follow-back behaviors do not correspond to the 3 patterns listed in the survey. Coincidently, they all indicate that they seldom or never clean up their followees (100%) as shown in the rightmost plot of Fig 2.

**Fig 3:**
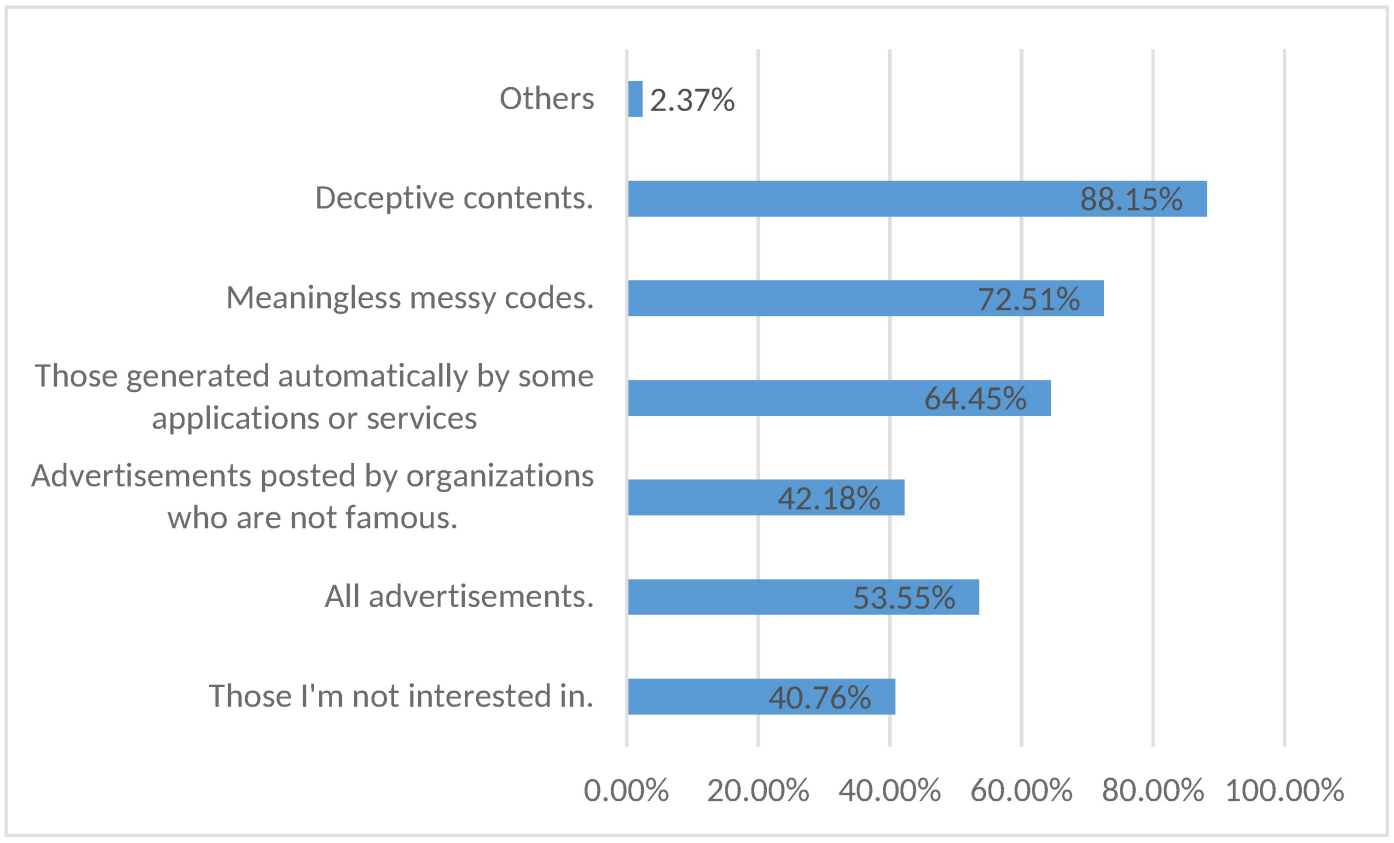
Users’ definition for low-quality content (Abstract categories).

**Fig 4:**
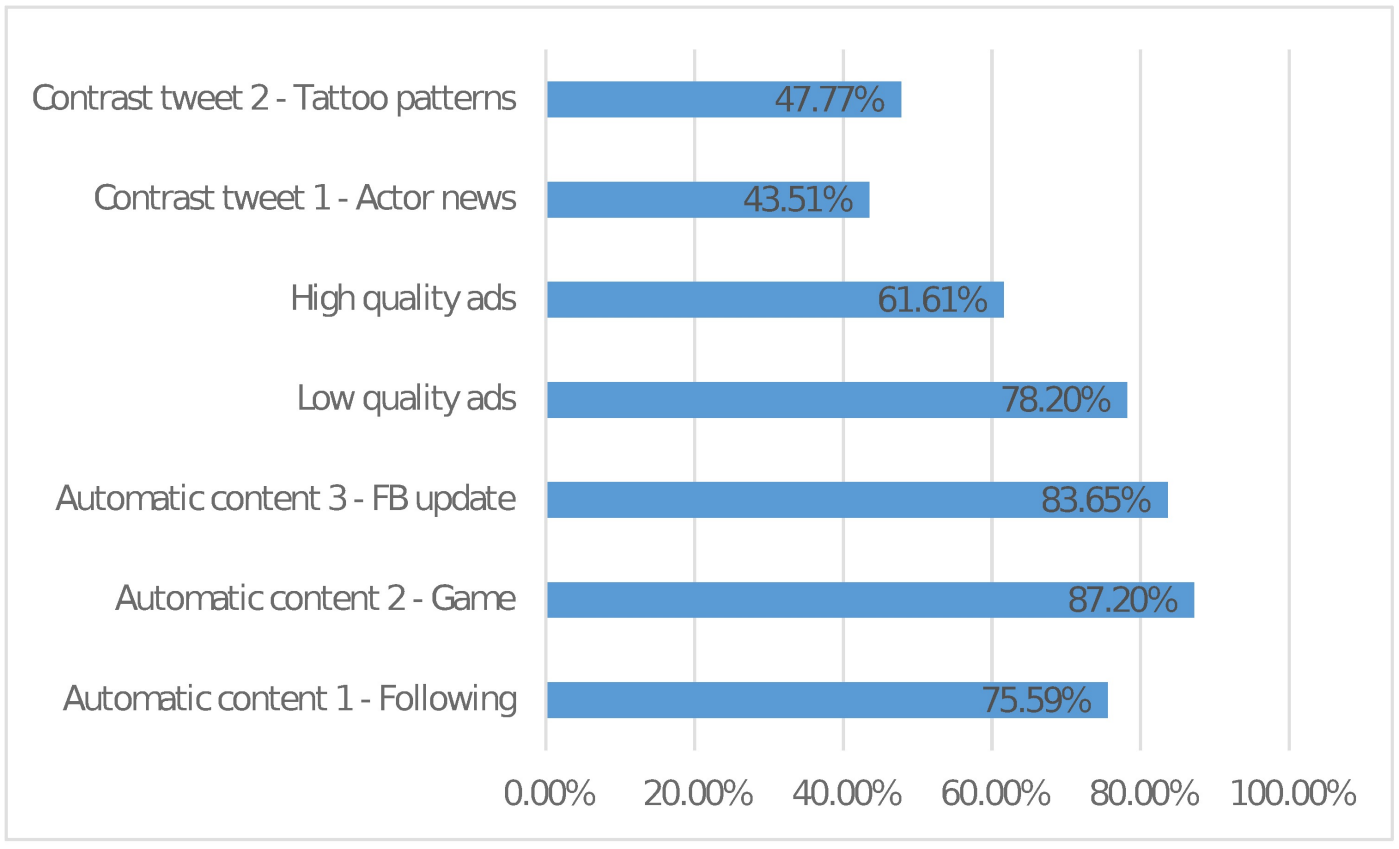
Users’ definition for low-quality content (Specific examples).

To investigate the users’ definitions for low-quality content, we provide abstract (general) categories in one question (see Fig 3) and specific example tweets in another question (see Fig 4). The two questions are designed according to the cluster analysis results in the previous phase. Participants are asked to tick what they regard as low-quality content.

It is generally agreed that deceptive content and messy codes belong to low-quality content but what is interesting here is that the category which receives the third most complaints is automatically generated content by some applications and services. Accordingly, two automatically generated tweets receive the most complaints by users as shown in Fig 4.

In Fig 4, it is observed that users feel more upset with low quality advertisements posted by obscure individuals or organizations rather than high quality advertisements posted by well-known individuals or organizations. Nevertheless, even high quality advertisements receive a large percentage of complaints (61.61%) from users which is consistent with the results shown in Fig 3 that 53.55% of users think all advertisements are low-quality content. Again, this underscores the fact that existing work mainly focuses on detecting malicious content and does not pay much attention to low-quality content which actually bothers users a lot.

We include two contrast tweets in the survey. Contrast tweets are those regarded as normal by the annotators in the preliminary studies. These contrast tweets are presented so that the survey respondents are being reminded of what normal tweets are and we expect less people will regard these contrast tweets as low-quality content compared to the real low-quality content. As seen in Fig 4, the two contrast tweets also receive more than 40% of the responses of being recognized as low-quality content and this may be due to the fact that 40.76% of the participants believe uninterested content should also be regarded as low-quality content.

Another factor observed that may affect users’ attitudes towards suspicious content is actually the proportion of low-quality content among all the tweets posted by a suspicious user. From Table 4, 19.43% of those surveyed will start considering unfollowing if low-quality content made up more than 25% of all tweets while 35.55% of them will start considering unfollowing if more than 50% were low-quality content.

**Table 4:** What’s the maximum threshold (as a percentage of your recently received messages) you can bear before considering unfollowing him/her?

Options	Number	Ratio
Too bothersome to unfollow	28	13.27%
Nearly 100%	15	7.11%
More than 75%	52	24.64%
More than 50%	75	35.55%
More than 25%	41	19.43%

Hence, from our previous work [40] and based on the results of our survey, we provide a clearer definition of low-quality content. We define it as a large amount of valueless or fraudulent content which hampers users from browsing useful content. They include low-quality advertisements, automatically generated content, meaningless content, click baits, etc. In other words, the “low-quality content” being referred to in this paper describes tweets instead of users which differentiates our work from existing research work which filter users [16] [17] [18] rather than the “offending” low-quality tweets. This is because some users post low-quality content **not** out of ill intention and on many occasions they are generated automatically by third-party applications previously authorized by the users according to our observations. These users may still post content which their followers are interested in so we simply cannot filter all users straightaway once they have ever posted low-quality content.

Moreover, according to the results shown in Table 4, a complement to the definition is that identical content posted by different users will not always get the same labeling results on whether they are low-quality content or not. This is because whether such content accounts for a large proportion of all tweets of that user is also an important factor. The frequency of such content determines the negative impact on overall user experience. If such content constitutes a huge proportion of a user’s tweets, they are regarded as low-quality content. If they are sporadic relative to a user’s total number of tweets, their followers will not mind them so much. Thus our definition of low-quality content is not necessarily an absolute unit of content but can be relative and vary from user to user.

## Identifying features characterizing low-quality content

Low-quality content detection is usually viewed as a classification task. A lot of features have been proposed for spam or phishing detection. The question about whether these features can be adopted for detecting the low-quality content defined in this paper will be addressed in the later section. In this section, we provide an in-depth analysis of features proposed by us and the common features presented in existing studies. We then determine the dominant features from the perspective of both time and accuracy for low-quality content detection.

### Direct features

The typical structure of a tweet crawled is in JSON format. All the information included in this raw JSON tweet can be directly extracted almost at the same time it is posted. These features are the most efficient ones in real-time low-quality content detection from the perspective of time performance. Since they can be extracted directly, they are called direct features (DF) in this paper. Direct features which can be extracted from the raw JSON tweet are listed in Table 5. Features 1 to 10 are Tweet based while the rest are profile based.

**Table 5:** Direct features.

Index	Feature	Comments
1	Source	Tweeting tools
2	Type	Regular, Replies, Mentions and Retweets.
3	Retweet_count	The number of times the tweet is retweeted.
4	Favorite_count	The number of times the tweet is favorited
5	Hashtags_count	The number of hashtags in the tweet.
6	Urls_count	The number of urls in the tweet.
7	Mentions_count	The number of mentions in the tweet.
8	Media_count	The number of media in the tweet.
9	Symbols_count	The number of cashtag in the tweet.
10	Possibly_sensitive	If the tweet possibly contains sensitive content.
11	Location	If the location field of profile is null.
12	URL	If the URL field of profile is null.
13	Description_len	The length of the description field of.
14	Verified	If the user is verified by Twitter.
15	Ff_ratio	Followers_count / Friends_count
16	Followers_count	The number of followers of the user.
17	Friends_count	The number of friends of the user.
18	Statuses_count	The number of statuses the user post.
19	Favourites_count	The number of tweets the user favorite.
20	Listed_count	The number of lists the user create.
21	Account_age	The lifespan of the account.
22	Default_profile	If the user is using a default profile.
23	Default_profile_image	If the user is using a default avatar.

Since a user can post multiple tweets, the profile based features for different tweets posted by the same user are identical while tweet based features may be different from tweet to tweet but can be the same for tweets posted by different users because of retweets.

### Indirect features

However, direct features alone cannot always give the best performance. According to the users’ responses presented in the previous sections, the proportion of low-quality content also affects users’ definitions for low-quality content. Thus indirect features (IF) are also identified. Indirect features are those which cannot be directly extracted from the crawled JSON tweet. Instead, a separate request is sent to Twitter to obtain the additional information. Indirect features capture the history information and tweeting behaviors of a user which will be proven to be significant for low-quality content detection in the later section. The purpose for adopting both direct and indirect features is to achieve a balance between detection accuracy and time performance.

The indirect features are listed in Table 6. As the indirect features are historical data of a particular user, most of them are profile based except for the last one. We are the first to use media, symbols and lists related features for similar detection tasks.

**Table 6:** Indirect features.

Index	Feature	Comments
1	Source_count	No. of sources used for posting n latest tweets.
2	Type_count	No. of types of the latest n tweets posted.
3	Hashtags_proportion	% of tweets with hashtags in the latest n tweets.
4	Urls_proportion	% of tweets with urls in the latest n tweets.
5	mentions_proportion	% tweets with mentions in the latest n tweets.
6	Media_proportion	% tweets with media in the latest n tweets.
7	Symbols_proportion	% tweets with symbols in the latest n tweets.
8	Sensitive_proportion	% tweets possibly sensitive
9	Nonfriends_interaction	If the tweet is an interaction between non-friends.

### Word level analysis

However, both direct and indirect features do not take the semantic meaning of the original tweet text into consideration. Thus word level analysis is designed to capture the content characteristics of the tweet text. Like spam emails, some keywords such as click, free are more frequently seen in low-quality content than in normal tweets.

Actually, word level analysis is frequently used in spam detection for emails [41] [42] [43] while not that popular in spam detection on OSN. Possible reasons may be the extensive use of informal abbreviations and the limited length of a tweet. [14] uses a wordpress comment blacklist but we suppose this blacklist may not be suitable for low-quality content detection on Twitter. Thus, in our studies, we analyze those tweets labeled as low-quality content and try to find the terms which happen most frequently and build a blacklist keyword dictionary by ourselves. We exploit the bag-of-word model to process the original 10,000 tweet texts. There is one word bag for low-quality content and another for normal tweets. Then we remove stop words in each bag. For terms in the word bag of low-quality content, the term frequency is used to represent the weight of the term but the weight will be reduced if the same word also appears in the bag of normal tweets. Then we sort the word in the bag of low-quality content according to their weight and the top N words make up the blacklist keyword dictionary. The selection of N will be discussed in the later section.

Each term in this dictionary can be viewed as one feature and these features together with the direct and indirect features proposed earlier are combined for detecting low-quality content. In the results and evaluation section, we will validate the efficacy of all direct and indirect features as well as the word level analysis proposed in this section.

What is worth mentioning here is that for a real time environment, the dictionary will evolve in order to capture the “hot words” in low-quality content. The update of the dictionary can be implemented in real time while the reconstruction of the corresponding training set should be done at regular intervals. A blacklist keyword dictionary with a size of 150 is shown in S2 Text. It is noted that it is not possible for such a dictionary to include all blacklist keywords and that is why it should be updated regularly. In addition, the purpose of such a dictionary is to help improve the detection performance of low-quality content instead of listing as many blacklist words as possible. Further details about the performance improvement are discussed in the later section.

## Pre-implementation tweet processing

### Data collection and preprocessing

To collect tweet data, we use one thread to crawl tweets through public streams provided by Streaming API. The tweet crawled in this way is in the JSON format. Another thread is run at the same time to parse the raw tweet and then extract the direct features shown in Table 5.

Twitter REST APIs provide access to read and write Twitter data such as posting a new tweet, reading author profile and follower data, etc. In our case, we use a third thread to send a request to function *statuses/user_timeline* so as to obtain the latest tweets of a particular user and calculate the corresponding indirect features listed in Table 6. The three threads can work simultaneously in order to save time for detection.

For the preparation of word level analysis, we exploited the *Text Mining (tm)* Package developed for R [44]. For tweets marked as low-quality content, we used regular expressions to remove all RT, @, # tags as well as all URLs in tweets. Then we preserved only English characters and transformed them to lower case. These tweets were then forwarded to the *tm* library to remove all stop words. One consideration here was whether we should stem these tweets after removing the stop words as the stemming step could help reduce the number of possible terms but with the risk of losing part of the word meanings. The details will be discussed in the results and evaluation section.

Our Twitter dataset consists of 100,000 tweets generated by 92,720 distinct users. These tweets are collected from 16th May to 17th May 2016. The days are randomly selected with no particular reasons. The reason we do not adopt a larger dataset is because in the following procedure we are going to label the dataset manually so as to verify the accuracy of our study results.

### Labeling tweets

To develop an automatic low-quality content detection system, it is necessary to build a training set. We have set up some label guides based on the survey results to ensure the label from annotators can fully convey users’ opinions. If the tweet falls into the four categories discussed in preliminary studies, the timeline of the user will also be considered. If similar low-quality content appears frequently (usually more than 50% of latest tweets posted) in the timeline of the user, the tweet will be labeled as low-quality content, otherwise we regard it as normal tweet. What should be noted here is that we do not label other tweets appearing in the timeline of the user, they are just regarded as a reference during the labeling process. In other words, they are not considered as labeled tweets.

We choose Cohen’s Kappa coefficient (*k*) to evaluate the inter-rater agreement of the labeling which is also used in [45] and [46] for similar purpose. Our annotation results reach a high agreement of *k* = 0.90

In total, we labeled the 100,000 tweets crawled based on both the original tweet and its user’s timeline, the data and the labels can be seen in S1 Table. Among these tweets, 9,945 of them are labeled as low-quality content.

### Training and testing classifiers

The focus of the evaluation is to show the feasibility of derived features in real-time detection of low-quality content. Hence the classification method used is not the focus. According to [33] and [47], Random Forest and Support Vector Machine outperforms other classifiers for detecting spam and phishing. Thus we choose the two classifiers to perform the low-quality content detection task. We train the classifier on the training set with a 5-fold validation. Then we perform the model on the test set and checked the prediction against the labeled results.

A series of experiments are conducted to evaluate the performance of our proposed low-quality content detection system. 100,000 labeled tweets are being used to test the system to gather the prediction results as well as to evaluate the computation time. All the experiments are run on a Dell Precision T3600 PC with Intel Xeon E5-1650 processor at 3.20 GHz with 16 GB of RAM.

## Implementation results and evaluation

### Word level analysis

To achieve a better performance through word level analysis, two special factors are discussed in this subsection. One is the size of the keyword blacklist dictionary. Usually a larger dictionary will increase the detection accuracy but may fall into the overfitting problem. For each word preserved in the low-quality content corpus, its weight determines whether it can be added into the dictionary. Its weight is represented by its term frequency in low-quality content minus its term frequency in normal tweets. We can vary the dictionary size by setting different thresholds for weight.

**Fig 5:**
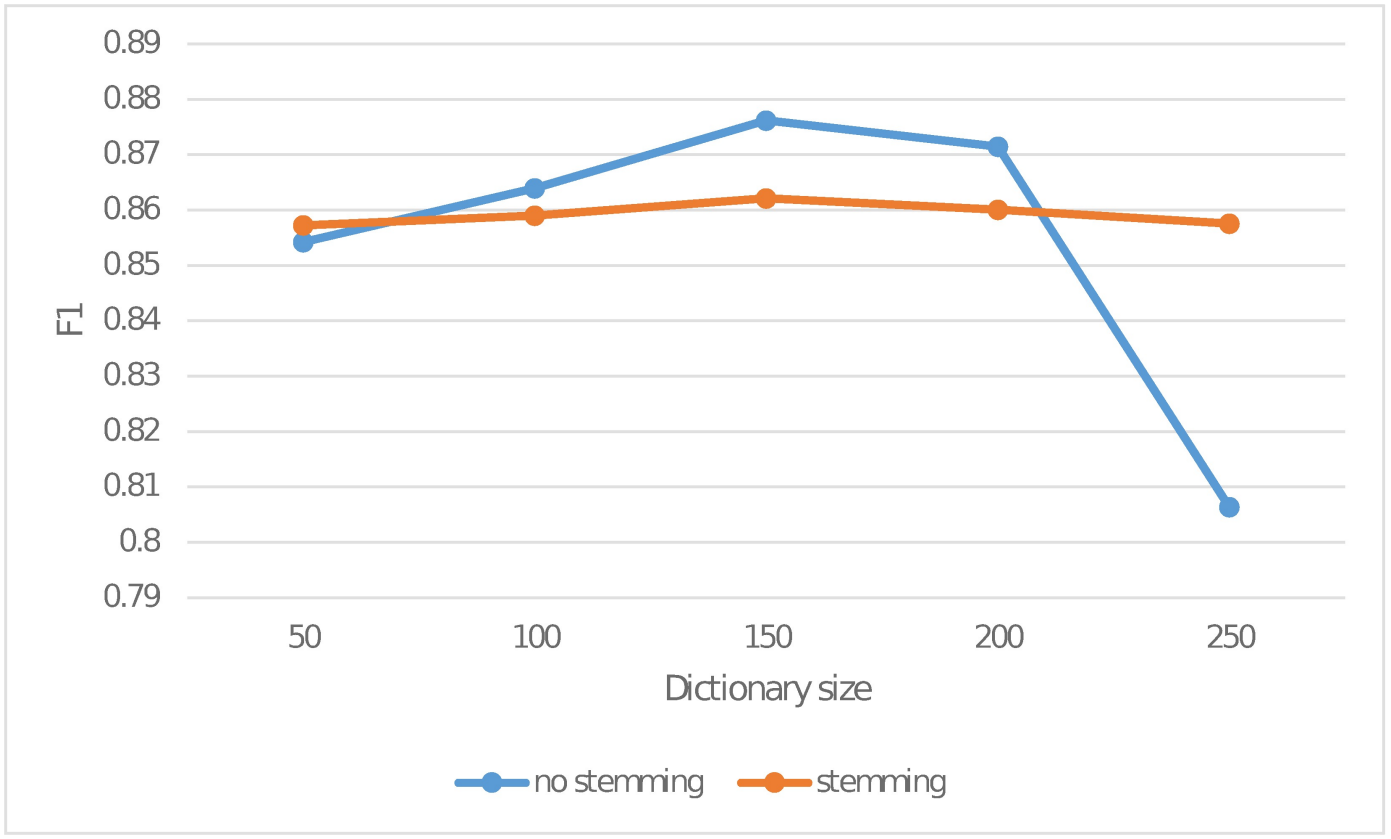
F1 measure with and without stemming.

The other controlled factor is whether to perform stemming on the tweet texts during the preprocessing phase. In this subsection, we perform low-quality content detection with different dictionary size and evaluate the performance from the perspective of both time and detection rate. The F1 measure results are shown in Fig 5.

It is observed from Fig 5 that when the size of the keyword blacklist dictionary becomes larger, the detection performance increases moderately. However, when the dictionary size is further increased, both of them fall into the trap of over-fitting. No stemming performs better than stemming when the dictionary size is not very large but experiences an early and severe drop in detection performance when dictionary size increases. Another advantage of no stemming is that it can save the time cost which will otherwise be incurred for the extra stemming step. According to our observations, we set the dictionary size to 150 and skip the stemming step in the following experiments.

### Feature rank

**Fig 6:**
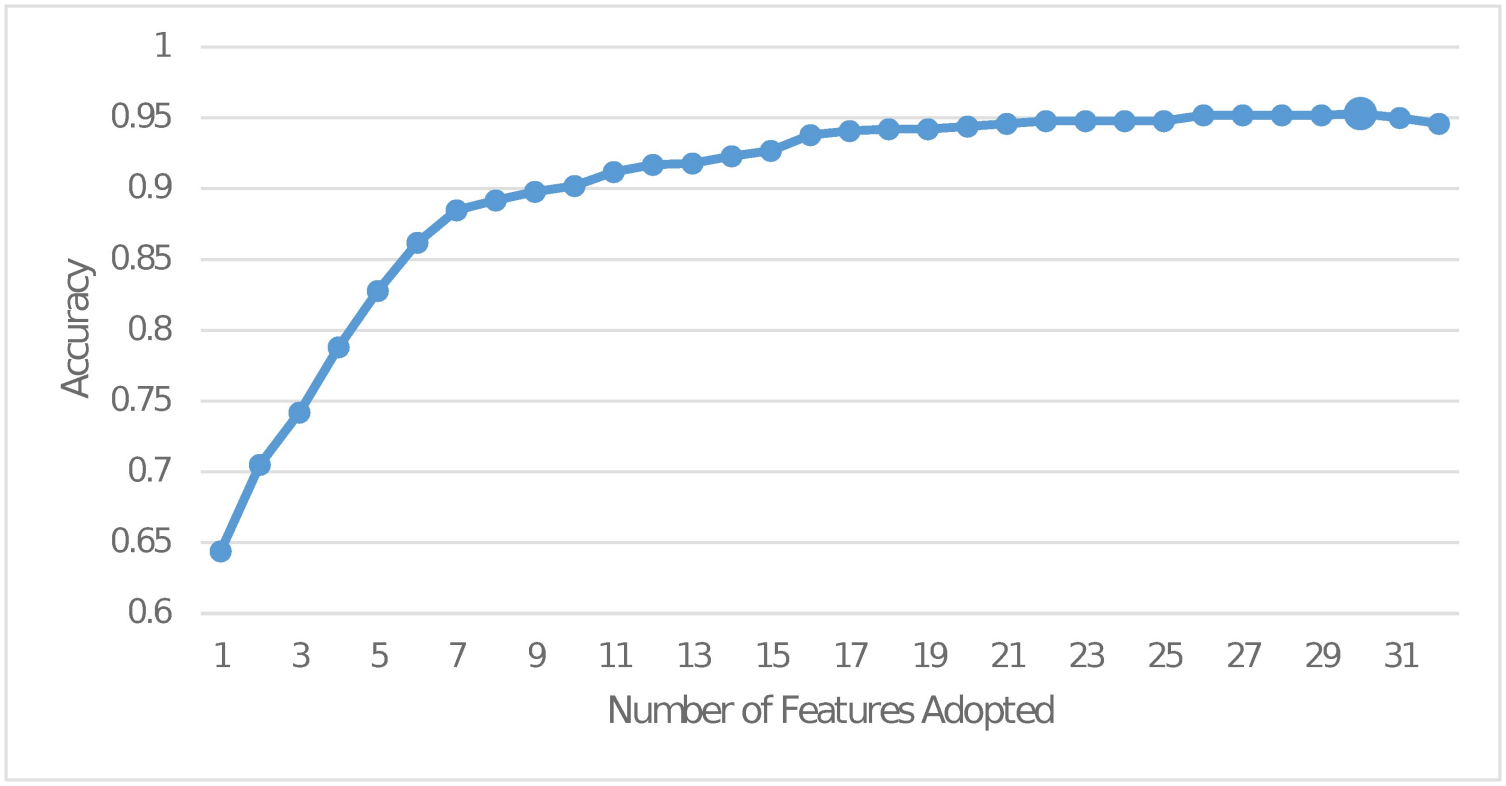
Accuracy of different subsets of features.

The construction of the keyword blacklist dictionary has already included the selection of important word features. In this subsection, we would like to discuss more about the significance of other direct and indirect features. Initially, we applied the Recursive Feature Elimination (RFE) to test the performance of using different subsets of features described before and the results are shown in Fig 6. It is observed that the accuracy reaches a peak when using 30 features out of a total of 32 features. This indicates most of the features we adopt are quite efficient for detecting low-quality content. What is worth mentioning here is that even when adopting only 10 features, the accuracy can reach more than 90%. The top 10 features selected by RFE can be seen in the last column of Table 7.

**Table 7:** Feature rank.

IG	CHI	AUC	RFE
mention_prop	mention_prop	favourites_count	follwers_count
url_prop	url_prop	type_cnt	friends_count
media_prop	media_prop	urls_cnt	statuses_count
type_cnt	favourites_count	url_prop	url_prop
favourites_count	type_cnt	mention_prop	listed_count
friends_count	friends_count	mentions_count	urls_count
urls_count	follwers_count	type	mention_prop
hashtag_prop	urls_count	default_profile	media_prop
follwers_count	hashtag_prop	ff_ratio	favourites_count
Type	type	hashtags_count	hashtag_prop

We also use three popular feature evaluation methods: Information Gain (IG), Chi-square test and Area under the ROC Curve (AUC) to compute the rank of the features and the top 10 features selected via different evaluation methods are shown in Table 7. It is to be noted that the focus is to extract the top ranking features. Hence, the relative quantitative performance is not shown.

The results show that most of the indirect features are more efficient in detecting low-quality content then direct features. This is because the indirect features also take the history data of a user into consideration. Among all the features, mention_prop, url_prop and favourites_count are selected by all four feature evaluation methods.

### Detection performance

In this subsection, we would like to provide more details to illustrate the performance of our proposed method for detecting low-quality content by adopting different subsets of features. According to the observed results in the last section, we set the dictionary size to 150. In our experiments, we have three subsets of features. Feature Subset I includes all direct features. Feature subset II includes all direct and indirect features. Feature Subset III includes all direct and indirect features plus word level analysis. We perform both Random Forest (RF) and Support Vector Machine (SVM) for the low-quality content detection task and the detection performance results are shown in Table 8.

**Table 8:** Detection performance of different feature subsets.

**Random Forest**				
Feature Subset	Acc	Fpr	F1	Time(s)
I	0.9526	0.0103	0.7124	0.0002
II	0.9599	0.0089	0.7634	1.9327
III	0.9711	0.0075	0.8379	1.9342
**SVM**				
Feature Subset	Acc	Fpr	F1	Time(s)
I	0.9335	0.003	0.4981	0.0003
II	0.9418	0.0074	0.6089	1.9328
III	0.9562	0.0037	0.7199	1.9343

It can be concluded that RF always performs better than SVM. Direct features alone can help detect roughly 95.26% of the low-quality content and the time performance is more than satisfying—almost as soon as the tweet is posted. When both direct and indirect features are adopted, the accuracy increases moderately to 95.99%. The detection accuracy soars to 97.11% when taking word level analysis into consideration and the F1 measure also increases significantly to 0.8379. For all 3 subsets of features, the false positive rate remains low at about 0.01.

For time performance, unlike [47], we do not include the time for building the training model as the training phase can be done out of band. In other words, the time performance is the detection time of content polluters and it includes the time required for extracting features as well as that for prediction. For the experiments, we basically run the detection for all the tweets in the user’s timeline. The time performance presented here therefore serves to provide an insight into the real time capability of our proposal.

The processing time for feature subsets II & III is longer than that of subset I. This is because the indirect features incur response time for the additional request to Twitter REST API. This fact notwithstanding, the current time performance for subsets II & III is still acceptable for real-time detection requirement (less than 2s). To summarize, the results show that our proposed features are not only time efficient but can also achieve a good detection rate.

### Comparisons with other methods

#### Blacklists and Twitter policy

Blacklists are often used for detecting phishing or spam. The biggest problem with blacklists is that there is always a time lag between the occurrence of these malicious content and the report to the blacklists. This problem makes blacklists less efficient to fulfill the real-time detection requirements. Moreover, most of the blacklists like Google Safe Browsing focus on phishing or malware and do not pay much attention to low-quality content. The focus of Twitter suspension policy is a bit different from the mentioned blacklists but still falls into the same trap. We check the low-quality content’s status one month later, and **60%** of them are still there. One possible reason for this phenomenon is that Twitter mainly focuses on content which breaks Twitter rules and pays less attention to other low-quality content. Even if they can detect such content, they may not filter them because of commercial reasons. Due to the lack of an effective real-time low-quality content detection method, users’ timeline is filled with low-quality content which hampers them from browsing other meaningful content. The method we propose in this paper tackles the problem in a holistic manner since the low-quality content which we detect covers valueless content of different types from the users’ perspective and include spam and phishing which are commonly covered by existing works. Hence, our method has been proven to be of great value to improve the overall user experience.

#### Other spam/phishing detection methods

The reason we do not need to distinguish among spam, phishing and low quality advertisements is because they share similar characteristics. Furthermore, from the perspective of users, they do not care what categories these low-quality content belong to. To improve overall user experience, our aim is to filter them regardless of their category. However, other research work either focuses on spam detection or phishing detection, so it is not that meaningful to compare our method with theirs because the purpose is different.

Nevertheless, to provide some insight into the performance of the proposed method for detecting low-quality content, we still select two related research work for comparison. One is [16] and the other is [14]. We implemented their methods and performed low-quality content detection on our dataset as described in the previous section and the results are shown in Table 9.

**Table 9:** Comparisons of different methods.

Method	Acc	FPR	F1
**Ours**	0.9711	0.0075	0.8379
**Wang’s**	0.9580	0.0056	0.7538
**Lee’s**	0.8514	0.0919	0.7025

For Lee’s method, a possible reason which may explain the low detection rate is that the detection method is designed based on accounts instead of tweets. The high false positive rate further indicates that some users who are classified as content polluters (i.e. spammers) also post normal content which his followers may be interested in. This proves that the detection for low-quality content is better to be carried out on a tweet level instead of an account level. For Wang’s method, their false positive rate is slightly lower than ours while the accuracy and F1 measure are much worse. This is because our method is specially designed for low-quality content detection while their detection is mainly focused on spam. In addition, some of the features used in Lee’s method cannot fulfill the real-time requirement and the time cost of our method is similar to Wang’s. The comparison results prove that our method achieves a good performance in both time and detection rate for low-quality content detection.

## Summary and future work

### Conclusions

In this paper, we propose a solution to address the problem of detecting low-quality content on Twitter in real time. We first derive a definition for low-quality content as large amount of repeated phishing, spam and low quality advertisements which hamper users from browsing normal content and erode the user experience. This definition is based on the outcomes of a survey targeting real users of online social networks and is thus proposed based on the users’ perspective. It is very necessary to detect these low-quality content in real time so as to improve user experience on OSN. We have performed a detailed study of 100,000 tweets and identified a number of novel features which characterize low-quality content. We provide an in-depth analysis of these features and validate the efficiency of using word level analysis for real-time low-quality content detection. The direct and indirect features can actually distinguish most of these low-quality content and the accuracy is about 95%. In addition, when word level analysis is adopted, the accuracy soars to 97.11% while still maintaining a low false positive rate (0.0075) and a good F1 measure (0.8379). The time needed to process all features proves feasible for real-time requirement. Through a series of experiments, we demonstrate that our method can achieve a good performance for real-time low-quality content detection for online social networks from the perspective of both detection rate and time. Our method addresses the low-quality content problem holistically since the low-quality content which we detect covers all valueless content from the perspective of users and include spam and phishing which are commonly covered by existing works. Our method is therefore of great value to the users not just in removing spam and phishing but also serves to improve the overall user experience in real time.

### Future work

It can be seen in the survey described above that 40.76% (See Fig 3) of the participants believe that all the content which they are not interested in should be filtered as low-quality content. This interesting discovery indicates the necessity and value of a content filter for disinterested content on online social networks. Thus in the future, we plan to add more customized configuration to the current work to implement a more personalized content filter not only focusing on general low-quality content. It is meant to automatically learn what the user is not interested in and hide them from the users’ timeline.

## Supporting information

S1 Text Survey about users’ opinions on low-quality content. This document contains the complete survey discussed in this paper about users’ opinions on low-quality content on Online Social Networks.
(DOCX)

S2 Text Examples of blacklist keywords. This document contains the blacklist keyword dictionary of a size of 150 words.
(DOCX)

S1 Table Tweet data and labels. This document contains the IDs of the tweets in our dataset as well as the labels.
(XLSX)

S1 Fig Screen captures of different categories of content polluters. This document contains the screen captures of the different categories of content polluters.
(DOCX)
