# Supplementary material for: A study on real-time low-quality content detection on Twitter from the users’ perspective
Source: PLoS One. 2017 Aug 9;12(8):e0182487. doi: 10.1371/journal.pone.0182487 (PMC5549928; doi:10.1371/journal.pone.0182487)
Supplement: S1 Text — This document contains the complete survey discussed in this paper about users’ opinions on low-quality content on Online Social Networks. (DOCX) [file pone.0182487.s001.docx]

**S1 Text. Survey about users' opinions on low-quality content.**

1. **How often do you use social network sites (e.g. Twitter, Facebook, Weibo, etc)? (Single choice)**

Nearly everyday.

At least once a week.

Less than once a week.

Seldom or never.

1. **How often will you clean up your followees/friends? (Single choice)**

Seldom or never.

More than once a month.

At least once a month.

Almost every week.

1. **If someone follows you, will you follow back? (Single choice)**

I usually follow back out of courtsey.

I only follow those I know.

I only follow those who share common interests with me.

I usually don't follow back.

1. **What will you regard as low-quality content when you are using social network sites? (Multiple choices)**

Those I'm not interested in.

All advertisements.

Advertisements posted by organizations who are not famous.

Those generated automatically by some applications or services (Not updated by users).

Meaningless messy codes.

Deceptive contents.

1. **Please tick the boxes of those you regard as content polluters. (Multiple choices)**

Today stats: One follower, No unfollowers via (URL omitted)

I've collected 7,715 gold coins! (URL omitted) #android, #androidgames, #gameinsight

I posted a new photo to Facebook (URL omitted)

Hot, my little pony friendship city light curtain .(hm118) - Full read by eBay (URL omitted)

New Toshiba Encore 7 16GB Intel Atom WiFi tablet - Full read by eBay (URL omitted)

Anupam Kher completes 31 years in Bollywood (URL omitted)

23 Clever Tattoos You Might Not Actually Regret In 50 Years (URL omitted)

1. **How much do content polluters affect your user experience when using social network sites? (Single choice)**

Very much.

A bit but still bearable.

A little.

They don't affect my user experience.

1. **What's the maximum threshold (as a percentage of your recently received messages) you can bear before considering unfollowing him/her? (Single choice)**

I don't care too much about content polluters and it's too bothersome to unfollow others.

Nearly 100%.

More than 75%.

More than 50%.

More than 25%.

1. **Are you willing to use an extension/application to help filter content polluters on social network sites? (Single choice)**

Yes.

No.

I'm not sure.
