# Supplementary material for: A study on real-time low-quality content detection on Twitter from the users’ perspective
Source: PLoS One. 2017 Aug 9;12(8):e0182487. doi: 10.1371/journal.pone.0182487 (PMC5549928; doi:10.1371/journal.pone.0182487)
Supplement: S2 Text — This document contains the blacklist keyword dictionary of a size of 150 words. (DOCX) [file pone.0182487.s002.docx]

**S2 Text. Examples of blacklist keywords.**

> dic

[1] "weather" "updates" "theweatherchannel" "channel" "transponder" "snail"

[7] "although" "automatically" "follow" "followed" "libra" "unfollowed"

[13] "practical" "tug" "aries" "profess" "capricorn" "conflicting"

[19] "checked" "virgo" "embol" "maintaining" "pragma" "prowess"

[25] "readily" "gemini" "scorpio" "sides" "apparent" "capable"

[31] "strategic" "foresee" "imagination" "unfolding" "approach" "measurable"

[37] "taurus" "comprehend" "stellar" "aquarius" "enables" "highly"

[43] "pisces" "det" "sagittarius" "leo" "emotions" "financial"

[49] "somethin" "fully" "follows" "understanding" "calm" "closest"

[55] "planning" "witness" "clearly" "convince" "found" "begin"

[61] "creative" "matters" "followers" "huaraches" "presentation" "sex"

[67] "attitude" "earning" "seem" "gucci" "cancer" "gain"

[73] "giants" "benefits" "checkout" "giveaway" "challenge" "encounters"

[79] "custom" "monsters" "tos" "coins" "pips" "wild"

[85] "collected" "current" "mgwv" "harvested" "candid" "changes"

[91] "enter" "pill" "retweet" "straw" "null" "bikini"

[97] "smart" "stats" "upskirt" "blowjob" "masturbation" "unfollowers"

[103] "bayonet" "followtrick" "mbf" "camel" "limitless" "anal"

[109] "hats" "click" "followback" "teamfollowback" "unf" "vagina"

[115] "anotherfollowtrain" "positively" "eurusd" "flashiest" "horny" "lesbian"

[121] "seems" "sexy" "adurabyhenshawblaze" "csgorumble" "fade" "mhmm"

[127] "safaree" "supporters" "allegedly" "amateur" "cock" "newborn"

[133] "samuels" "thumb" "alubarna" "facial" "healthier" "milf"

[139] "reflective" "useless" "wers" "badboy" "baths" "bbw"

[145] "busty" "decay" "loaner" "oral" "pussy" "sail"
