# Supplementary material for: A study on real-time low-quality content detection on Twitter from the users’ perspective
Source: PLoS One. 2017 Aug 9;12(8):e0182487. doi: 10.1371/journal.pone.0182487 (PMC5549928; doi:10.1371/journal.pone.0182487)

**S1 Fig. Screen captures of the different categories of content polluters.**

What should be noted here is that the screen captures are collected about one year after the original work, some of the content polluters have been deleted by Twitter. In order to give the reader a general idea of different categories of content polluters, we collect some of them recently.

1, Low quality advertisements:


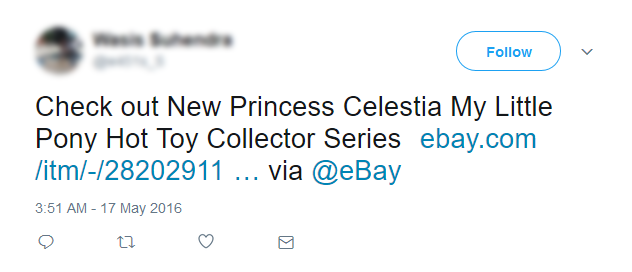

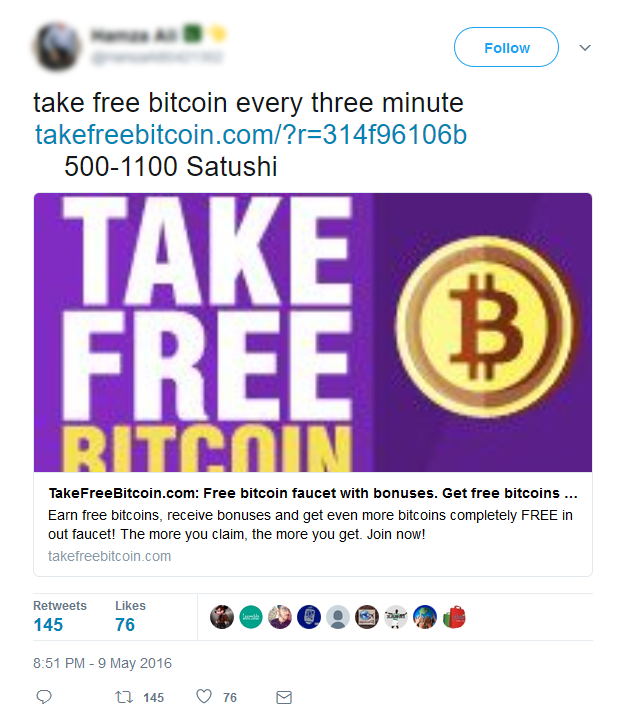


2, Automatically generated content


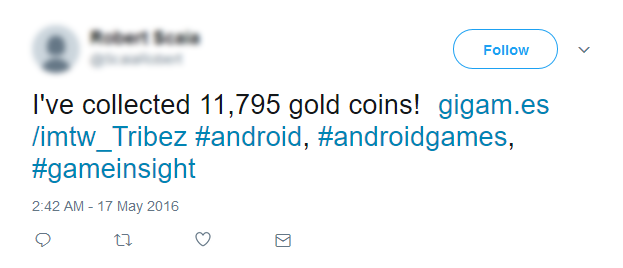

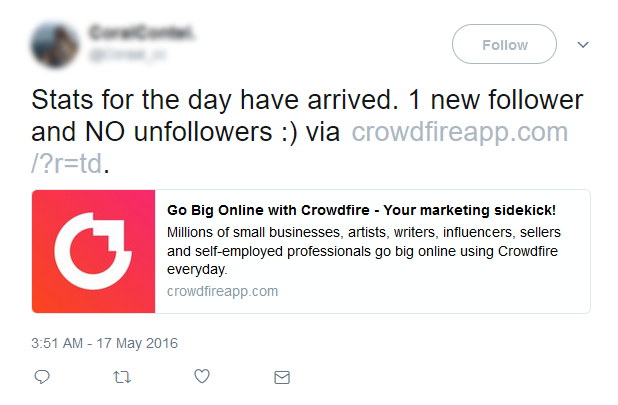


3, Meaningless content


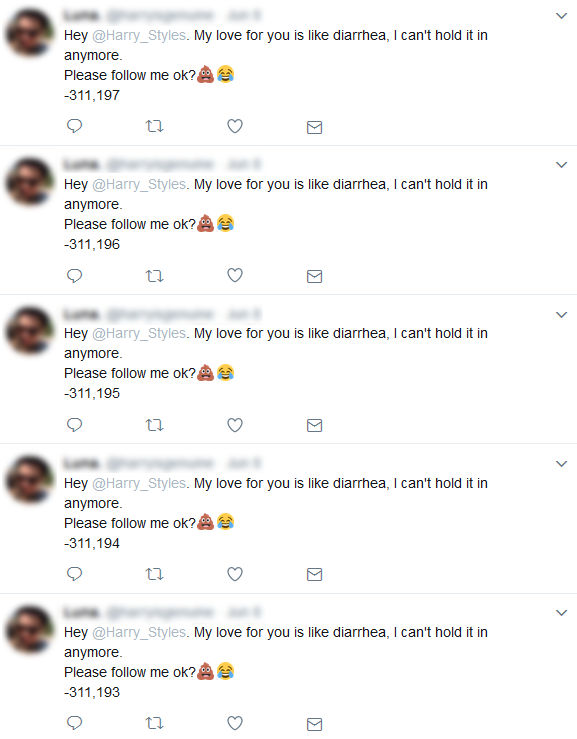

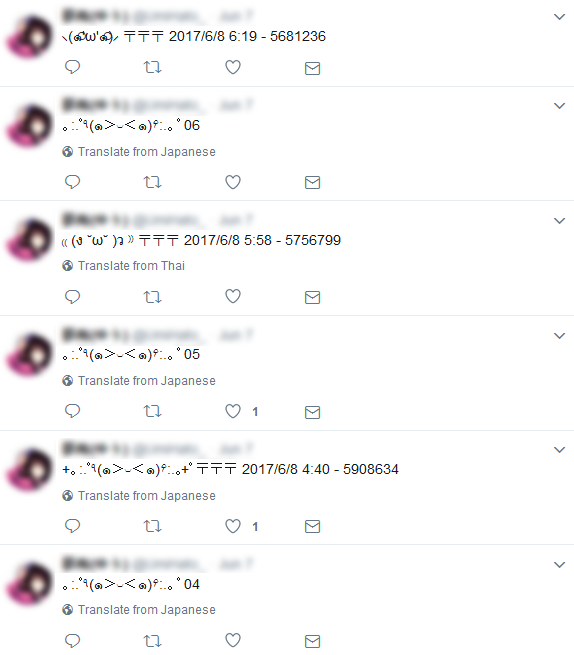

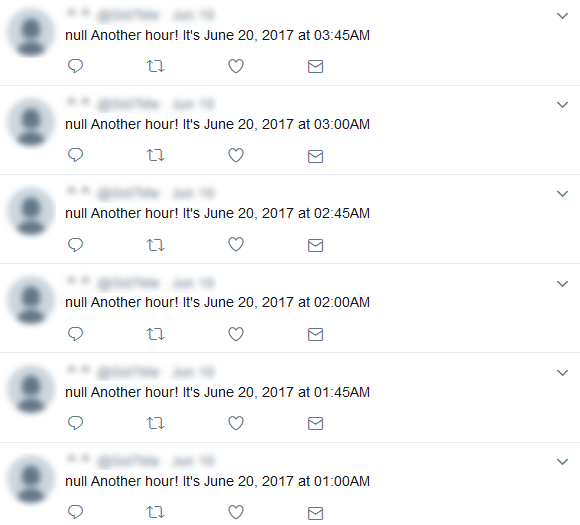


4, Click baits


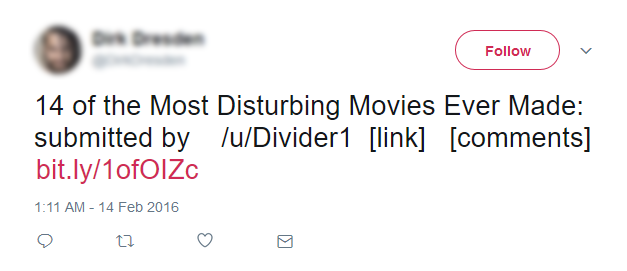

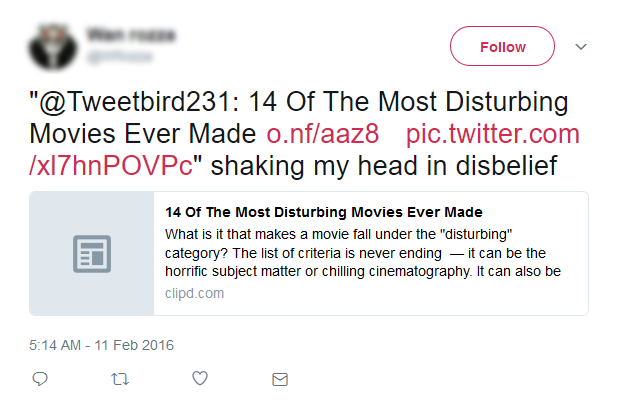

Supplement: S1 Fig — This document contains the screen captures of the different categories of content polluters. (DOCX) [file pone.0182487.s004.docx]
